# The influence of a modified lipopolysaccharide O-antigen on the biosynthesis of xanthan in *Xanthomonas campestris* pv. campestris B100

**DOI:** 10.1186/s12866-016-0710-y

**Published:** 2016-05-23

**Authors:** Tim Steffens, Frank-Jörg Vorhölter, Marco Giampà, Gerd Hublik, Alfred Pühler, Karsten Niehaus

**Affiliations:** Proteom- und Metabolomforschung, Fakultät für Biologie, Centrum für Biotechnologie (CeBiTec), Universität Bielefeld, Universitätsstraße 27, 33615 Bielefeld, Germany; Genomforschung industrieller Mikroorganismen, Centrum für Biotechnologie (CeBiTec), Universität Bielefeld, Universitätsstraße 27, 33615 Bielefeld, Germany; Jungbunzlauer Austria AG, Pernhofen 1, 2064 Wulzeshofen, Austria; Present address: MVZ Dr. Eberhard & Partner, Brauhausstr. 4, 44137 Dortmund, Germany

**Keywords:** *Xanthomonas campestris*, Xanthan, Exopolysaccharide, LPS, O-antigen, Phytopathogen

## Abstract

**Background:**

The exopolysaccharide xanthan is a natural product which is extensively used in industry. It is a thickening agent in many fields, from oil recovery to the food sector. Xanthan is produced by the Gram negative bacterium *Xanthomonas campestris* pv. campestris (Xcc). We analyzed the lipopolysaccharide (LPS) of three mutant strains of the Xcc wild type B100 to distinguish if the xanthan production can be increased when LPS biosynthesis is affected.

**Results:**

The Xcc B100 O-antigen (OA) is composed of a linear main chain of rhamnose residues with N-acetylfucosamine (FucNAc) side branches at every second rhamnose. It is the major LPS constituent. The O-antigen was missing completely in the mutant strain H21012 (deficient in *wxcB*), since neither rhamnose nor FucNAc could be detected as part of the LPS by MALDI-TOF-MS, and only a slight amount of rhamnose and no FucNAc was found by GC analysis. The LPS of two other mutants was analyzed, Xcc H28110 (deficient in *wxcK*) and H20110 (*wxcN*). In both of them no FucNAc could be detected in the LPS fraction, while the rhamnose moieties were more abundant than in wild type LPS. The measurements were carried out by GC and confirmed by MALDI-TOF-MS analyses that indicated an altered OA in which the branches are missing, while the rhamnan main chain seemed longer than in the wild type. Quantification of xanthan confirmed our hypothesis that a missing OA can lead to an increased production of the extracellular polysaccharide. About 6.3 g xanthan per g biomass were produced by the Xcc mutant H21012 (*wxcB*), as compared to the wild type production of approximately 5 g xanthan per g biomass. In the two mutant strains with modified OA however, Xcc H28110 (*wxcK*) and Xcc H20110 (*wxcN*), the xanthan production of 5.5 g and 5.3 g, respectively, was not significantly increased.

**Conclusions:**

Mutations affecting LPS biosynthesis can be beneficial for the production of the extracellular polysaccharide xanthan. However, only complete inhibition of the OA resulted in increased xanthan production. The inhibition of the FucNAc side branches did not lead to increased production, but provoked a novel LPS phenotype. The data suggests an elongation of the linear rhamnan main chain of the LPS OA in both the Xcc H28110 (*wxcK*) and Xcc H20110 (*wxcN*) mutant strains.

**Electronic supplementary material:**

The online version of this article (doi:10.1186/s12866-016-0710-y) contains supplementary material, which is available to authorized users.

## Background

*Xantomonas campestris* pv. campestris (Xcc) is a Gram negative, phytopathogenic, γ-proteobacterium. It is the causative agent of the black rot disease in cruciferous crops including all cultivated brassicas [1–3]. Furthermore it is the producer of the exopolysaccharide xanthan [4].

The Xcc B100 genome was published in 2008 [5] and was used to reconstruct the biosynthetic pathway for xanthan production. Xanthan is an anionic heteropolysaccharide, and its production depends on the export and polymerization machinery formed by the products of the *gum* gene cluster that includes 12 genes from *gumB* to *gumK* [6]. Xanthan is of extensive industrial usage as a thickening agent for many applications, such as in food, oil drilling and in the cosmetics industry [7]. It consists of pentasaccharide repeat units formed by a backbone of two glucose moieties and a side chain formed by two mannose and one glucuronic acid residues attached to position 3 at every second glucose. Acetate and pyruvate groups are non-stoichiometrically bound to the mannose residues [7]. Xanthan production originates from glucose 6-phosphate and fructose 6-phosphate, which are both crucial molecules in the central metabolism and can easily be linked to nucleotides to form nucleotide sugars [5, 8]. The nucleotide sugars GDP-mannose, UDP-glucose and UDP-glucuronic acid are the direct precursors for xanthan production. The synthesis takes place at the inner membrane of Xcc, where each repeating unit is bound to a lipid carrier, most likely undecaprenyl phosphate. Following transition to the outer face of the cell membrane, the repeating units are polymerized and the xanthan molecule is exported through pore proteins [5, 7]. Other saccharides next to xanthan, such as the lipopolysaccharides (LPS), also depend on nucleotide sugars as precursors. LPS are the most common molecules in the outer leaflet of the outer membrane of Gram negative bacteria and are important for maintaining the structural stability of these cells. In Xcc, several studies have revealed the structure and determined biological functions for the LPS [9–14]. In other *Xanthomonas* species the composition of this gene cluster differs fundamentally from Xcc, possibly as a consequence of horizontal gene transfer events [15–18]. In general LPS consists of three parts. Lipid A is the membrane anchor and is built from two glucosamine molecules, to which fatty acids are bound. The fatty acids can be bound as esters or as amides. Furthermore, the length and number of the fatty acids may vary [19, 20]. In Xcc, phosphate is attached at position 4 of the first, and at position 1 of the second, glucosamine. The second part of the LPS is the core oligosaccharide. It is covalently bound to the Lipid A and in Xcc it consists of one 3-deoxy-D-*manno*-octulonsonic acid (Kdo), three mannose, and one glucose residues. In addition, galacturonic acid moieties can be linked to the Kdo and to the innermost mannose via phosphate molecules [10]. The third and largest part of the LPS is the O-antigen (OA). It is exposed into the surrounding medium and in Xcc it consists of trisaccharride repeating units formed by a backbone of two rhamnose molecules and a N-acetyl fucosamine (FucNAc), which is covalently bound to the second rhamnose [11]. Much like the biosynthesis of xanthan repeating units, OA repeating units are supposed to be synthesized on membrane bound undecaprenyl phosphate lipid carriers [20]. For *X. hortorum* pv. vitians it was reported that the rhamnan backbone is synthesized independent of the branches and that FucNAc therefore is additionally added after the main chain synthesis [21]. While LPS are usually regarded to be indispensable for the bacterial cell [19], the three LPS main components vary in their relevance for cell viability. The OA is the longest and the most exposed part of the molecule, but it is not essential for the bacteria in order to maintain structural stability and survive.

Since both, xanthan and LPS, are linked in their production at the level of nucleotide sugar metabolism, it could be possible to redirect such precursor molecules to xanthan biosynthesis. Also mutants, later identified as Xcc OA mutants, were described with increased colony mucoidity, which may indicate an improved exopolysaccharide production [14, 22]. Hence, three mutant strains of Xcc roughly known to be affected in the OA were chosen to more precisely determine the LPS phenotypes and to analyze a possible effect on xanthan production. The genes responsible for OA biosynthesis are organized in a 15 genes comprising cluster called *wxc* [14]. Both, the complete OA and the FucNAc branches were targeted in this approach. The results reveal new insights in the nucleotide sugar metabolism with respect to xanthan production and LPS biosynthesis, respectively. Furthermore, we found evidence for an altered O-antigen structure in mutants with inhibited OA branches.

## Results and discussion

### The genetic background of three *X. campestris* pv. campestris B100 strains carrying mutated genes involved in the biosynthesis of the LPS O-antigen

**Fig. 1:**
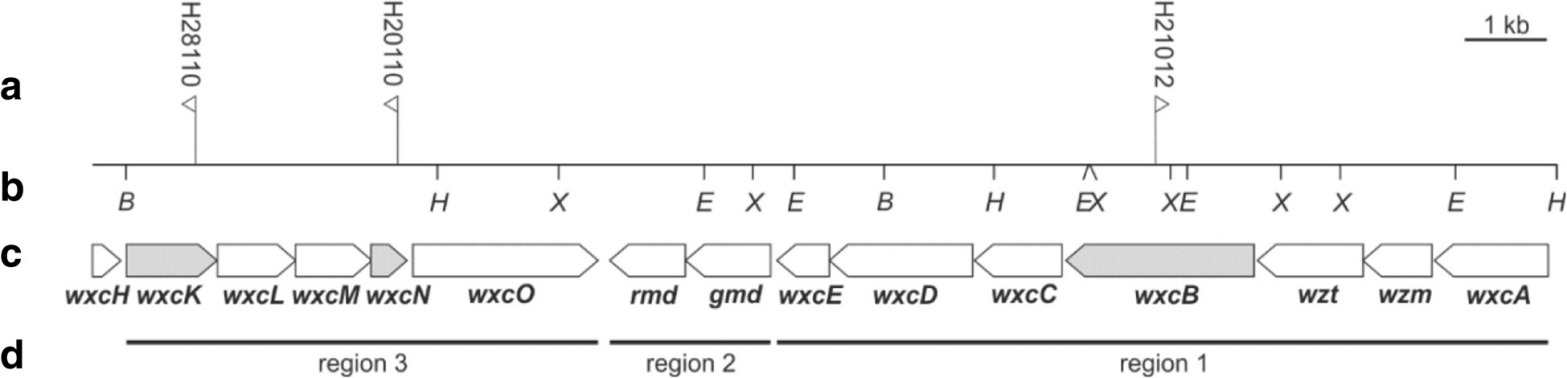
Gene region involved in the LPS O-antigen biosynthesis in *Xanthomonas campestris* pv. campestris B100. **a** Mutant names (Xcc H21012 (*wxcB*), Xcc H28110 (*wxcK*) and Xcc H20110 (*wxcN*)) and positions of Tn5 insertions in the Xcc the genome are marked [14, 22]. The flag indicates the orientation of the *lacZ* reading frame. **b** Restriction sites of endonucleases are given (B: *Bam*HI; E: *Eco*RI, H: *Hin*dIII; X: *Xho*I). **c** Detailed map of the *wxc* gene cluster with the respective gene names. Mutated genes are highlighted in grey. **d** Functional organization of the gene cluster following the analyses of Vorhölter and colleagues [14]: Region 1 is supposed to be involved in the biosynthesis of the poly-rhamnan main chain, region 2 plays a role in the nucleotide sugars biosynthesis leading to GDP-rhamnose. Region 3 is putatively responsible for the N-acetylfucosamine branches of the Xcc OA

**Fig. 2:**
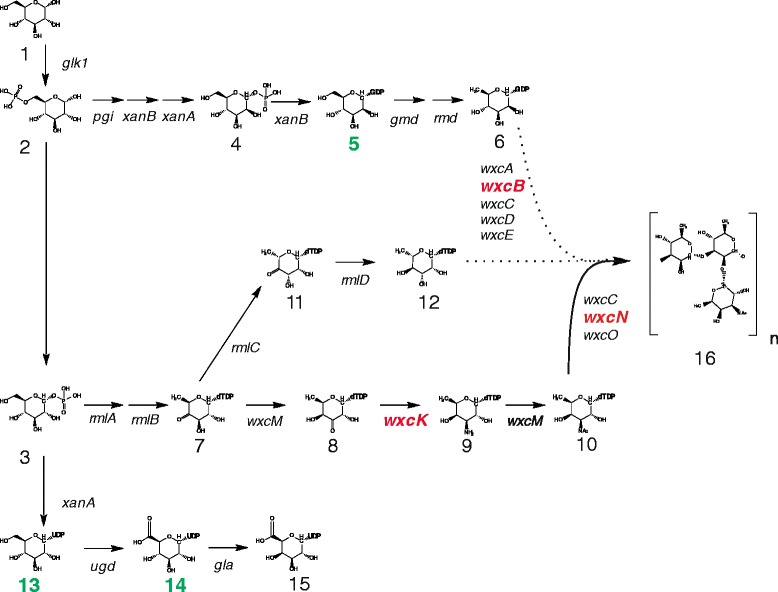
Relevant part of the *Xanthomonas campestris* pv. campestris sugar nucleotide metabolism producing precursors for the synthesis of the LPS O-antigen and the exopolysaccharide xanthan. Enzymatic reactions are indicated by arrows and names of the involved genes. Mutated genes of the strains Xcc H21012 (*wxcB*), Xcc H20110 (*wxcN*) and Xcc H28110 (*wxcK*) are indicated in bold red letters at their respective position within the metabolic pathway. Molecules are numbered as followss: 1: D-Glucose; 2: D-Glucose 6-phosphate; 3: D-Glucose 1-phosphate; 4: D-Mannose 1-phosphate; 5: GDP-D-mannose; 6: GDP-D-rhamnose; 7: dTDP-4-keto-6-deoxy-D-glucose; 8: dTDP-3-keto-6-deoxy-D-galactose; 9: dTDP-3-amino-3,6-dideoxy-D-galactose (dTDP-D-fucosamine); 10: dTDP-*N*-acetyl-fucosamine; 11: dTDP-4-keto-6-deoxy-L-mannose; 12: dTDP-L-rhamnose; 13: UDP-D-glucose; 14: UDP-D-glucuronic acid; 15: UDP-D-galacturonic acid; 16: OA repetitive subunit, dashed lines indicate uncertainties regarding the OA biosynthesis from rhamnose precursors. The xanthan precursors (5, 13, 14) are marked in green

Genes located in the *wxc* gene cluster are responsible for the LPS O-antigen in Xcc. The cluster had been identified by sequence analysis of the cosmid pXCB1002 [22], followed by an initial analysis of the LPS phenotypes of mutant strains where genes were interrupted by Tn*5*-*lacZ* transposon or interposon insertions [14, 22]. Functions had been attributed to most of the genes within the *wxc* gene cluster. Three distinct regions were distinguished (Fig. 1). Region 1 consists of seven genes essential for the biosynthesis of the O-antigen in Xcc [14]. The products of the two genes in region 2 are involved in the nucleotide sugar metabolism [14]. For the genes in region 3, functions had only been annotated based on sequence analysis and no LPS species that differ from the wild type profile could be detected for analyzed mutants. The annotation indicated, however, that the gene products of region 3 were putatively involved in the biosynthesis and transfer of a N-acetyl-3-amino hexose [14]. As N-acetylfucosamine (FucNAc) was subsequently identified as part of the Xcc O-antigen [11] we concluded that proteins encoded by genes of region 3 might be involved in the biosynthesis of the O-antigen branches. A branched metabolic pathway was reconstructed that compiles information regarding nucleotide sugar biosynthesis in Xcc (Fig. 2).

So far it has not been analyzed whether mutations affecting LPS biosynthesis have an effect on xanthan production even though the biosynthesis of both molecules is closely connected, as both, xanthan and LPS molecules depend on the same precursor pool of nucleotide sugars [5, 14].

We analyzed the mutant strains Xcc H21012 (*wxcB*)*,* Xcc H28110 (*wxcK*) and Xcc H20110 (*wxcN*) that were constructed by Hötte et al., 1990 [22] as Tn*5*-*lacZ* insertion mutants [23]. Polar effects of these mutations cannot be excluded, however for the purposes of this study it is not essential but should be mentioned. The aim of this study was to analyze inhibiting effects of the OA synthesis and to assess whether its inhibition would be a feasible target to improve xanthan production.

The mutant Xcc H21012 (*wxcB*) was chosen since its initial characterization indicated a complete inhibition of the OA biosynthesis [14]. The gene *wxcB* is located in region 1 of the *wxc* cluster (Fig. 1). Genes from this genomic region are necessary for the biosynthesis of the poly-rhamnan main chain of the OA [14] (Fig. 2). While the precise function of *wxcB* was still obscure, it has been described as an essential gene for OA biosynthesis in Xcc with a putative kinase function [14]. Additionally, a recent study in *X. euvesicatoria* [24], which is still frequently addressed by its legacy name *X. campestris* pv. vesicatoria, predicts WxcB to be involved in cellular motility, cell wall and membrane biosynthesis, furthermore the mutant had an increased biofilm production [25], whereas xanthan is an important part of the biofilm [26, 27], while no effects on LPS could be detected when *wxcB* is affected by mutation [25]. The phenotype for the *wxcB* mutant H21012 was shown previously by Vorhölter et al. [14] and even polar effects affecting the following genes of a transcriptional unit in *wxc* cluster region 1 would not change the subject of this study, might however explain why no effect in the *X. euvesicatoria* LPS was detected [25]. Genes from region 3 are not part of the transcriptional unit of region 1 genes, therefore polar effects affecting region 1 and region 3 genes can be excluded, as they also show different OA patterns [14]. It can, however, not be excluded that both mutants, Xcc H28110 (*wxcK*) and Xcc H20110 (*wxcN*), show similarities due to polar effects of the Tn5 insertions. Still, both mutated genes have an assigned putative function for the branching of the Xcc OA, but data on LPS level were missing [14]. Therefore analysis of both mutants was needed, since the aim was not to elucidate individual gene functions, but to analyze the impact of putative OA branch mutants.

The gene *wxcK* is reported to encode an enzyme of the metabolic pathway responsible for dTDP-FucNAc biosynthesis [14]. The nucleotide dTDP-FucNAc is the putative precursor for the FucNAc branch of the Xcc OA (Fig. 2), but in the initial characterization no LPS phenotype could be detected in a corresponding mutant [14]. Inhibition of *wxcK* could have an effect on xanthan production, since xanthan precursors might be gained through the inhibition of dTDP-FucNAc. Similar to *wxcK*, *wxcN* is a gene located in region 3 of the *wxc* cluster (Fig. 1). It codes for a small membrane protein that is putatively involved in the branching of the OA, possibly with the transfer of FucNAc residues [14] (Fig. 2). However, until now no function could clearly be assigned to *wxcN,* also no LPS phenotype has been detected so far [14]. Moreover the impact on xanthan production was uninvestigated.

The experimental procedure followed these considerations to determine the impact of LPS mutations on xanthan production, furthermore, the LPS phenotypes were determined.

### Biochemical analysis of three *X. campestris* pv. campestris B100 mutant strains revealed a missing O-antigen for Xcc H21012 (*wxcB*) and a modified O-antigen for Xcc H20110 (*wxcN*) and Xcc H28110 (*wxcK*)

**Fig. 3:**
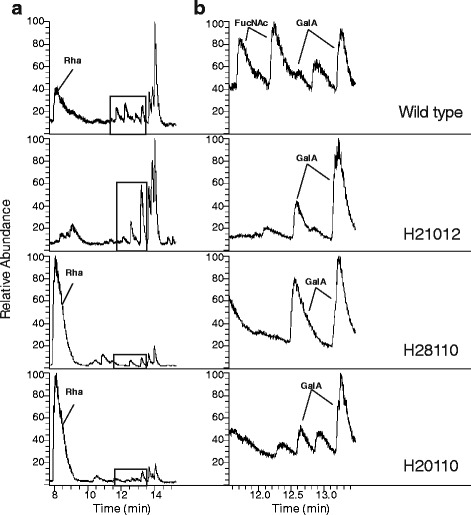
Analysis of the LPS O-antigen of *Xanthomonas campestris* pv. campestris B100 wild type and three mutant strains by gas chromatography following methanolysis. Wild type data are compared to the *wxcB* mutant H21012, *wxcK* mutant H28110, and *wxcN* mutant H20110. **a** Complete chromatogram with the O-antigen main constituent rhamnose (Rha) annotated followed by a box indicating signals related to the second O-antigen constituent N-acetylfucosamine (FucNAc) and LPS core moieties. **b** Zoom-in of the boxed area of the chromatogram with peaks identified to represent the O-antigen constituent FucNAc and the LPS core constituent galacturonic acid (GalA), which are both represented by two peaks. Measurements were carried out after LPS isolation applying the hot phenol / water method followed by methanolysis with 0.5 M HCl in methanol and the respective peracetylation of 100 μg LPS

**Fig. 4:**
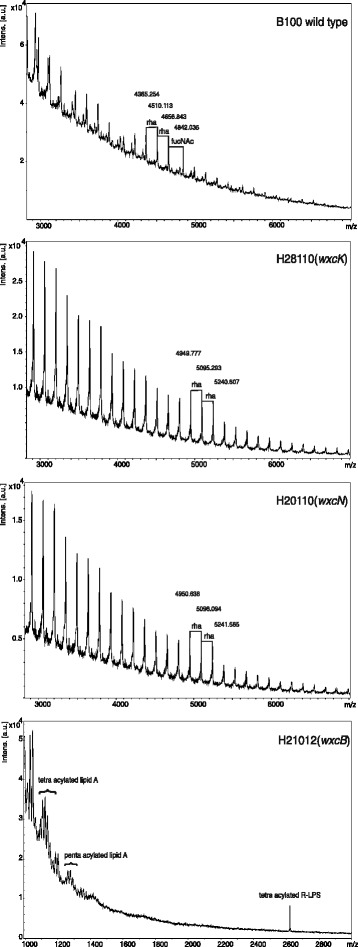
MALDI-TOF-MS analysis of the LPS O-antigen of *Xanthomonas campestris* pv. campestris B100 wild type, the O-antigen branch mutants Xcc H28110 (*wxcK*) and Xcc H20110 (*wxcN*) and O-antigen deficient mutant Xcc H21012 (*wxcB*). Spectra related to the mutants Xcc H28110 (*wxcK*) and Xcc H20110 (*wxcN*) to the B100 wild type O-antigen were compared in order to verify and deeper analyze the inhibition of the OA side branches and the increase in rhamnose moieties. Displayed are ranges of the spectra with a focus on the O-antigen constituents, rhamnose (rha) and N-acetylfucosamine (FucNAc). Examples of repeating peaks are marked. No OA residues were detected in the LPS of H21012 (*wxcB*), but we could measure different lipid A moieties and a peak representing the rough-type LPS. Measurements were carried out with 5 μg LPS of each strain

In order to determine the phenotype for the *Xcc* OA, LPS isolation with the hot phenol / water method described by Westphal and Jann [28] was performed after cultivation of the bacteria in shaking flasks with complex medium. Complex medium was used, since the xanthan production in minimal medium hinders LPS isolation. GC analysis followed after methanolysis and peracetylation of the isolated LPS, furthermore MALDI-TOF-MS was performed on isolated LPS (Fig. 3, Fig. 4). In a study by Vorhölter et al., 2001 [14] the mutant LPS had been briefly characterized by SDS-PAGE. While in SDS-PAGE only the Xcc H21012 (*wxcB*) mutant had a visible phenotype, GC and MALDI-TOF analyses are suitable to reveal structural details which cannot be resolved by gel-electrophoresis. Hence, we applied these techniques to re-assess the previously inconspicuous results of the mutants Xcc H20110 (*wxcN*) and Xcc H28110 (*wxcK*) LPS [14].

By GC-MS analysis of wild type LPS, a peak for rhamnose and a double peak for N-acetyl fucosamine were detected, both representing the *Xcc* OA monosaccharide constituents [11]. The OA components eluted from the GC column prior to the core moieties of galacturonic acid, mannose, and glucose (Fig. 3). To further validate the results obtained by GC-MS we performed MALDI-MS measurements with hot phenol / water isolated LPS. The obtained mass spectra prove an OA comprised of both, rhamnose and FucNAc (Fig. 4).

The GC analysis of mutant strain Xcc H21012 (*wxcB*) had no peaks for rhamnose nor for N-acetyl fucosamine, while the LPS core constituents were present (Fig. 3). Thus, data clearly show the missing of the complete OA, corresponding well with the results of the initial SDS-PAGE characterization of the mutant [14]. These findings were validated by MALDI-MS measurements of Xcc H21012 (*wxcB*) LPS (Fig. 4). Furthermore this MALDI measurement was used to assign peaks representing the Xcc lipid A, as well as a rough-type LPS, since no OA constituents were interfering with these ions. Both the lipid A moieties, as well as the rough-type LPS, were assigned to masses resulting from calculations with compounds detected in the initial structural determination of the Xcc LPS [10]. For the lipid A moieties we were able to detect two different acylated forms, a tetra- and a penta-acylated lipid A and also heterogeneity within the acylation pattern could be shown (Fig. 4). The lipid A fragments were present as ion groups that showed a lipid A composition of different fatty acids, which is well known for the lipid A and in accordance with the structural determination by Silipo et al. [10].

The GC chromatogram of the Xcc H28110 (*wxcK*) mutant was a surprise. While no LPS phenotype had been observed when the mutant LPS had been initially characterized by SDS PAGE [14], the FucNAc signal observed for the wild type was absent in H28110 LPS, indicating an inhibition of the OA side branches biosynthesis. However, as compared to the wild type chromatogram we observed an enlarged rhamnose peak, indicating a vast increase of the rhamnose constituents of the OA main chain (Fig. 3). This corresponds to the inconspicuous results of the SDS-PAGE analysis of the mutant LPS [14] as such a structure variation is not visible by gel electrophoresis-based analysis. The mass spectrum resulting from the MALDI-TOF-MS analysis has a very regular pattern of peaks that differ by a mass of 145–146 Da, which represents the OA main chain constituent rhamnose (Fig. 4). This further validates the previous GC analysis, since no FucNAc was detected and the peak areas indicate a high abundance of rhamnose moieties in the Xcc H28110 (*wxcK*) LPS.

The results obtained when the LPS of the mutant strain Xcc H20110 (*wxcN*) was analyzed had some similarities to the results described for the mutant Xcc H28110 (*wxcK*). Here too, the SDS-PAGE analysis had indicated an inconspicuous LPS phenotype [14]. The GC chromatogram of Xcc 20110 (*wxcN*) had an enlarged rhamnose moiety peak, while no FucNAc was detected (Fig. 3). The MALDI-MS spectra of both mutants looked very much alike, with regular peaks that differ in 145–146 Da, representing rhamnose (Fig. 4). These findings again support the GC chromatogram of Xcc H20110 (*wxcN*) LPS.

**Fig. 5:**
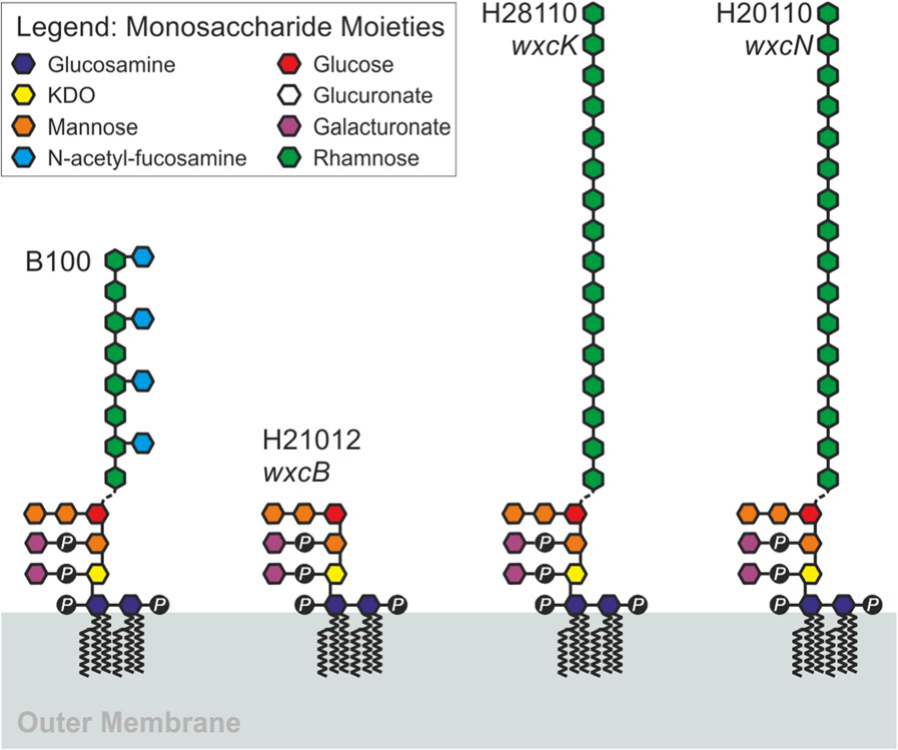
Model of putative LPS structures of the *Xanthomonas campestris* pv. campestris B100 wild type and the mutants Xcc H21012 (*wxcB*), Xcc H28110 (*wxcK*) and Xcc H20110 (*wxcN*). GC data indicate an elongation of the O-antigen rhamnose chain in both branchless mutants, implying that the FucNAc branches play a role in O-antigen chain length determination. The specific linkage between the rough-type LPS to the O-antigen is still unclear and thus shown as a dashed line. A graphical legend indicates the individual monosaccharide moieties

Furthermore, to get better insights in the amounts of OA constituent rhamnose, we performed a hydrolysis of the LPS followed by reduction of the sugars and peracetylation with the addition of 15 μg of xylose as an internal standard. The amounts of rhamnose could be determined, after we determined a response factor of rhamnose as compared to the same amount of xylose. The obtained GC chromatograms are depicted in the Additional files 1 and 2 and the calculated results are shown in Table 1. With rhamnose amounts of 255.8 μg and even 403.6 μg in Xcc H28110 (*wxcK*) LPS and Xcc H20110 (*wxcN*) LPS, respectively, the previously described results were strikingly confirmed, since in the Xcc B100 wild type LPS 80.6 μg of rhamnose was detected. This means more than three times as much rhamnose as in the wild type was detected in the *wxcK* mutant and even five times more in the *wxcN* mutant (Table 1). Moreover, we could calculate the percentage of rhamnose in the used LPS (Table 1). For the Xcc wild type 16 % of the total LPS was rhamnose, in the H28110 (*wxcK*) mutant 51.5 % of the LPS, and in H20110 (*wxcN*) even 80.5 % of the total LPS was rhamnose. Surprisingly rhamnose signals could be detected in the LPS of Xcc H21012 (*wxcB*). A total amount of 45.5 μg representing 9 % of the used sample was observed, equal to approximately half as compared to the rhamnose amounts of the Xcc B100 wild type. However, we still argue that no OA is present in the LPS of the *wxcB* mutant H21012. This is confirmed by the data obtained by methanolysis of the LPS (Fig. 3) and especially by the MALDI-MS. Otherwise OA fragments would appear in the MALDI spectra, like in the Xcc B100 spectra (Fig. 4). This is clearly not the case, so the rhamnose in the Xcc H21012 (*wxcB*) sample might actually not originate from the LPS itself, but for example from intermediate precursors which were isolated along the LPS. A possible explanation would be that the synthesis of OA rhamnose backbones may not be inhibited, but later the rhamnan chain cannot be transferred onto the rough-type LPS. Still, based on the obtained results, a model describing the structure of the LPS was established for the three analyzed mutants compared to wild type LPS (Fig. 5). Data gained by GC and essentially by MALDI measurements show the inhibition of the Xcc OA in Xcc H21012 (*wxcB*) resulting in the LPS model shown in Fig. 5, which supports the phenotype observed before [14]. Therefore we argue that *wxcB* as part of *wxc* region 1 is indeed essential for LPS OA production. Despite some primary structure similarity of the *wxcB* gene product to the N-terminal and central methyltransferase and kinase domains of *E. coli* O9 WbdD [29], the function of WxcB is still obscure. Different results were published for *X. euvesicatoria*, where no difference in LPS between a *wxcB* mutant and the wild type was detected in a gel-electrophoresis based analysis [25]. However, since we cannot exclude a polar effect of the Tn5 insertion mutant this could also be an explanation for the differences. Furthermore, there are also fundamental differences in the repertoire of LPS biosynthetic genes between *Xcc* and *X. euvesicatoria* [30]. Hence it is unclear whether the specific role of *wxcB* is similar in Xcc and *X. euvesicatoria*.

**Table 1:** Analysis of the O-antigen constituent rhamnose of *Xanthomonas campestris* pv. campestris B100 wild type and three mutant strains

Strain	Rhamnose (μg)	Percentage of rhamnose in the total LPS (%)	Rate of Rhamnose as compared to Xcc B100
Xcc B100	80.6	16	1
Xcc H21012 (*wxcB*)	45.5	9	0.56
Xcc H28110 (*wxcK*)	255.8	51.5	3.17
Xcc H20110 (*wxcN*)	403.6	80.5	5.01

Wild type data were compared to the *wxcB* mutant H21012, *wxcK* mutant H28110 and *wxcN* mutant H20110. Results were obtained by gas chromatography following hydrolysis and reduction of 500 μg LPS each. The depicted amounts are the mean of two replicates. For the *wxcB* mutant H21012 only one measurement could be used

Xcc H28110 (*wxcK*) is a mutant where the biosynthesis of dTDP-FucNAc is putatively inhibited. This nucleotide sugar should be the precursor for the OA branches, so no N-acetylfucosamine should be present in the LPS and indeed no FucNAc could be detected in the LPS analyses of the *wxcK* mutant (Fig. 3, Fig. 4). Nonetheless, a vast increase in rhamnose was detected for the OA of Xcc H28110 (Fig. 3, Fig. 4, Table 1). This indicates a novel LPS phenotype where the absence of the OA branches leads to an increase of rhamnose moieties in the main chain (Fig. 5). The loss of FucNAc in the LPS of the *wxcK* mutant clearly indicates the involvement of dTDP-FucNAc as a precursor for the OA branches. This novel LPS phenotype was confirmed by a second mutant, Xcc H20110 (*wxcN*). The chromatogram again showed a vast increase in rhamnose moieties, as compared to the wild type, while N-acetylfucosamine was only detected in traces (Figs. 3 and 4, Table 1), indicating the same phenotype for both mutants from *wxc* gene cluster region 3. Although the similar phenotype of both mutants could be due to polar effects, the results strongly support the hypothesis that region 3 is indeed essential for the FucNAc branches in the Xcc OA [14] and the developed LPS model for region 3 mutations (Fig. 5).

In summary, both strains with deficiencies in the OA side branch, the mutant with a defect in the biosynthetic pathway towards dTDP-FucNAc Xcc H28110 (*wxcK*) [14] and Xcc H20110 (*wxcN*), where the transfer of the FucNAc residue onto the rhamnose backbone is blocked [14], show very similar LPS structures and compositions. This indicates that the rhamnan main chain in Xcc is synthesized independently from the branches, as described for *X. campestris* pv. vitians [21]. These findings lead to the conclusion of an elongation of the OA backbone in the absence of FucNAc. LPS chain length regulation is still under debate, with a proposed variable geometry model by King et al., 2014 [31]. They describe a model based on the abundance of two proteins, WbdA and WbdD which clearly explains their findings in *E. coli* O9 OA length distribution. However, our findings indicate that for Xcc B100 the FucNAc branches are important in the determination of the OA chain length. The *E. coli* O9 OA is not branched like the OA from Xcc B100, and therefore the model of King and colleagues might not be applicable or has to be extended for branched OA or at least for the Xcc OA. Nevertheless, more research has to be done to elucidate the LPS chain length regulation of *X. campestris*. Next to the LPS analysis, xanthan production was of major interest since the focus of the study is on the nucleotide sugar metabolism of Xcc.

### Xanthan production of *X. campestris* pv. campestris B100 mutants characterized by a missing or modified O-antigen

**Fig. 6:**
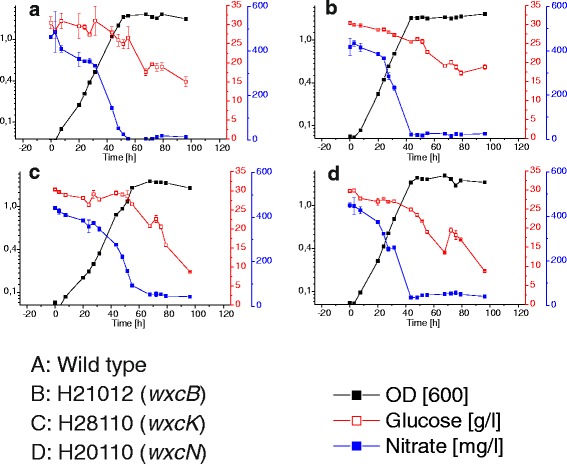
Cultivation of the *Xanthomonas campestris* pv. campestris B100 wild type (**a**) the three mutants Xcc H21012 (*wxcB*) (**b**), Xcc H28110 (*wxcK*) (**c**) and Xcc H20110 (*wxcN*) (**d**). All strains were grown in XMD minimal media with 0.6 g/l KNO_3_ as nitrogen source and supplemented with 30 g/l glucose as carbon source, at 30 °C and 180 rpm. The cultivation occurred simultaneously under the same conditions for 96 h. Displayed are the culture titer (OD), glucose and nitrogen consumption over time

The Xcc strains were cultivated in shaking flasks and simultaneously under the same conditions for all strains, at 30 °C and 180 rpm, in XMD minimal media containing 0.6 g per l nitrate as nitrogen source and 30 g per l glucose as carbon source to investigate effects of the LPS OA deficiencies on xanthan production. Strains were cultivated for 96 h and the results for one example cultivation are shown in Fig. 6, while others can be seen in the Additional files.

The cultivated wild type cultures stopped growing and started to enter the stationary culture phase after around 50 h with OD values at 600 nm of around 1.5 – 1.7 (Fig. 6, Additional file 3) and a mean growth rate of 0.053 h^−1^ (Additional file 4). During the whole cultivation the Xcc B100 wild type consumed 15–19 g*l^−1^ of glucose. Hence, glucose was clearly not a limiting growth factor (Fig. 6) since substantial amounts of glucose remained available as a resource for xanthan biosynthesis during the stationary phase. However, nitrate consumption was inversely correlated to the culture growth. Growth came to an end upon exhaustion of nitrate in the medium. In the Xcc wild type cultivations nitrate was consumed after approximately 48 h (Fig. 6).

During cultivation of the OA mutant Xcc H21012 (*wxcB*) cultures started to enter the stationary phase after around 44 h with OD values at 600 nm of around 1.6 – 1.8 (Fig. 6, Additional file 3) and with a mean growth rate of 0.062 h^−1^ it shows enhanced growth behavior (Fig. 6, Additional file 4). In comparison to the wild type, Xcc H21012 (*wxcB*) used with 10 to 13 g*l^−1^ less glucose. Like in the wild type cultivation, nitrate was the limiting growth factor. In the *wxcB* mutant it was consumed after around 44 h, which was faster than in the wild type cultivation and which was in agreement with the accelerated growth (Fig. 6).

The OA side branch mutant Xcc H28110 (*wxcK*) had the slowest growth (Fig. 6) in comparison to all other strains. Only after around 67 h optical densities of 1.7 – 1.9 were reached and the mean growth was 0.043 h^−1^ (Additional file 4). Although the growth was slower, the maximum OD values were not decreased. Interestingly we observed that Xcc H28110 (*wxcK*) used more glucose than the Xcc B100 wild type. In the cultivation approaches the mutant consumed more than 20 g*l^−1^ (Fig. 6, Additional file 3), once even all of the 30 g*l^−1^ initially present (Additional file 3). The nitrate consumption however, followed the culture growth and was slower than in the other cultures, while it was mostly consumed after around 67 h (Fig. 6, Additional file 3).

The growth of the second OA side branch mutant Xcc H20110 (*wxcN*) differed from Xcc H28110 (*wxcK*) and was even better than in the B100 wild type. The stationary phase was reached after approximately 50 h and the mean growth rate was 0.059 (Additional file 4). The H20110 (*wxcN*) mutant reached the stationary phase much like Xcc B100 after around 50 h, but the OD values reached up to 2 (Fig. 6, Additional file 3). Remarkably, the glucose consumption behaved more like the consumption in Xcc H28110 (*wxcK*), since more than 20 g*l^−1^ of glucose were used by every culture (Fig. 6, additional file 3). Most of the nitrate was used after around 44 h, again indicating the beginning of growth arrest (Fig. 6).

**Fig. 7:**
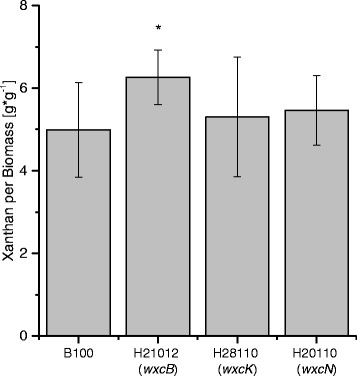
Xanthan production of the *Xanthomonas campestris* pv. campestris B100 wild type and the *wxcB* mutant H21012, the *wxcK* mutant H28110 and the *wxcN* mutant H20110. Production was measured as dry weight [g] xanthan per [g] biomass. Samples were harvested from ten replicates each. Harvest was after 96 h within the late stationary phase. Bars give the normalized mean xanthan yields, with error bars indicating the standard deviations

Moreover, differences in xanthan production could be detected, indicating a varied usage of the sugar nucleotide precursor pool (Fig. 7). Production was measured as dry weight [g] xanthan per [g] biomass. Ten replicates each were used to test for differential xanthan production and to minimize the hindering effect on sample handling due to the samples viscosity (Additional file 5). Harvest was after 96 h at the late stationary phase, in order to measure all of the produced xanthan. The xanthan production for of H21012 (*wxcB*) was clearly increased as compared to the Xcc B100 wild type. With a production of around 6.3 g xanthan per g biomass an increase of more than 1 g xanthan per g Xcc biomass was gained (Fig. 7).

In the branchless OA mutants Xcc H28110 (*wxcK*) and Xcc H20110 (*wxcN*) no relevant xanthan increase was measured as compared to the wild type. The wild type and Xcc H20110 (*wxcN*) produced between 5 and 5.5 g xanthan per g biomass. Xcc H28110 (*wxcK*) produced around 5.3 g xanthan per g biomass (Fig. 7). We then performed a *t*-test in order to check for significance of the differential xanthan production as compared to the Xcc B100 wild type (Additional file 5). The *p*-value of the xanthan production of the H21012 (*wxcB*) mutant as compared to the wild type was 0.03, which represents a significant increase in xanthan production. On the other hand the mutants Xcc H28110 (*wxcK*) and Xcc H20110 (*wxcN*) show *p*-values of 0.94 and 0.42, respectively. This clearly shows no significance in production as compared to the Xcc wild type.

Both branchless mutants, Xcc H28110 (*wxcK*) and Xcc H20110 (*wxcN*), consumed more glucose than the wild type. However, the xanthan production was rather unaffected. In contrast, in the Xcc H21012 (*wxcB*) mutant the glucose consumption was lower than in the wild type, despite an increased xanthan production. This indicates a differential usage of glucose in the mutants, as compared to the wild type. The data indicate that nitrate is indeed the limiting growth factor during cultivation. It is known that xanthan is mainly produced during the stationary phase, when nitrogen supply has come to an end and there is still a carbon source available [32]. We observed that while xanthan production was active in the stationary phase in all of the mutants and in the wild type, the productivity was different. Xcc H21012 (*wxcB*) was an efficient xanthan producer with nitrate consumption by 3 to 8 h faster than the wild type. A similarly increased nitrate consumption was observed for Xcc H20110 (*wxcN*), which however had no increased xanthan production. Overall, we can summarize that nitrate limited the growth. However, the remaining glucose was not only entirely used for xanthan production, but for other processes as well. In order to determine every process involved in glucose consumption, more research has to be performed, although a maintenance coefficient of 0.33 mmol per g and h was established for metabolic modeling in Xcc [33, 34]. In any case, the results obtained for the WxcB-deficient mutant strain Xcc H21012 indicate the potential of optimizing xanthan production by inhibiting competing metabolic pathways like the OA biosynthesis.

### The influence of a missing or a modified LPS O-antigen on the xanthan biosynthesis in *X. campestris* pv. campestris B100

The results for Xcc H21012 (*wxcB*) are in agreement with findings for *X. euvesicatoria* where an increase in biofilm production for a *wxcB* mutant was reported [25], as xanthan is an essential part of the *Xanthomonas* biofilm [26]. In addition, our results indicate a link between LPS and xanthan biosynthesis, since the inhibition of the OA led to an increased xanthan production. A reason for the increased production may be a redirection of nucleotide sugars towards xanthan biosynthesis since they are not needed for OA biosynthesis. Also, the availability of undecaprenyl phosphate lipid carrier, which are thought to be important factors for both xanthan and LPS production [5, 7, 20], might be improved and beneficial because they are not blocked by the nucleotide sugars required for OA biosynthesis. Still, increased biofilm and xanthan production might also be explained by the change of the bacterial surface, since no OA is present in the LPS. Additionally, the detection of decreased motility in Xcc H21012 (*wxcB*) (data not shown) that was also reported for *X. euvesicatoria* [25] might support the increase in xanthan production. Concurrence of decreased motility and increased biofilm production was also reported for the inhibition of the *shk*S histidine kinase gene of *Variovorax paradoxus* [35]*,* while a general connection between cell motility and biofilm production was observed for *Escherichia coli* and *Pseudomonas aeruginosa* [36].

To explain the unremarkable xanthan phenotypes of Xcc H20110 (*wxcN*) and Xcc H28110 (*wxcK*), one could consider the detected LPS phenotypes of the mutant strains. Putative reasons for enhanced xanthan production are the availability of a large nucleotide sugar pool, but in the OA branch mutants more nucleotide sugars seem to be used for a longer than usual OA main chain. Another reason for improved xanthan production could be a compromised bacterial surface, but as compared to the OA deficient strain Xcc H21012 (*wxcB*), both cases do not apply. Still, as the biosynthesis of LPS in xanthomonads is not well understood it is hard to identify mechanisms that are involved in synthesizing LPS with increased amounts of the OA main chain constituent rhamnose at the expense of the side branch constituent FucNAc, which was assumed to be affected by deficiencies either in the generation of dTDP-FucNAc precursors or in the FucNAc transfer to the growing OA main chain. The addition of the FucNAc side branches might be a limiting step of OA biosynthesis, so that the enzymes involved in synthesizing the OA main chain could operate at higher turnover numbers, resulting in OA with an increased number of rhamnose moieties when OA is synthesized without side branches. The branched biosynthetic pathway that delivers the LPS precursor molecules (Fig. 2) might point to an alternative explanation. The OA main chain precursor molecule dTDP-rhamnose and the side branch precursor dTDP-FucNAc are both derived from dTDP-glucose via the common intermediate dTDP-4-keto-6-deoxy-D-glucose. A block in the synthesis or transfer of dTDP-FucNAc might feed back to increase the metabolic pools of dTDP-4-keto-6-deoxy-D-Glc and dTDP-glucose, thereby providing additional precursors for the final reactions of dTDP-rhamnose biosynthesis. A comprehensive analysis of the Xcc LPS biosynthesis might provide data suitable to falsify such hypotheses, but would be a substantial endeavor due to the manifold and often challenging analysis techniques required to distinguish, identify, and quantify the many intermediate carbohydrate molecules involved that range from hexoses to oligo- and polysaccharides with fatty acid constituents.

## Conclusions

The analysis of three mutant strains with defects in the biosynthesis of the OA constituent of the Xcc B100 LPS revealed altered LPS OA for all mutant strains. Inactivation of *wxcB* resulted in a complete loss of the OA. Growth and viability of the mutant were unimpaired. Mutations affecting the genes *wxcK* and *wxcN* eliminated the FucNAc moieties that constitute the side branches of the OA. This was the first experimental evidence for an involvement of *wxc* region 3 genes in the biosynthesis of the LPS FucNAc constituents that so far had been postulated only on the basis of careful genome data interpretation [14]. An impact of inactivating the OA side branch biosynthesis on the OA main chain suggested that the OA branches may play a role in the main chain length regulation. Furthermore, the analysis of the *wxcB* mutant revealed a positive effect on the yield of xanthan. This opens a door for rationale design to increase xanthan production by reducing the drain of metabolic resources toward competing anabolic pathways.

## Methods

### Strains and cultivation

All strains used in this study are listed in Table 2. Bacteria were cultivated at 30 °C and 180 rpm in shaking flasks. Pre-cultures of Xcc and cultures for LPS isolation were grown in TY rich media [37] (5 g tryptone, 3 g yeast extract, 0.7 g CaCl_2_, per l), intermediate and main cultures in XMD minimal media [33], supplemented with 30 g/l glucose and 0.6 g/l KNO_3_ as nitrogen source, pH was adjusted to 7. When necessary, antibiotics were used in the following concentrations: Streptomycin (Sm): 800 μg/ml, kanamycin (Km): 80 μg/ml. Growth rate (RGR) was determined using the following equation:

**Table 2:** Bacterial strains used in this study

Strain	Relevant features	Reference
*X. campestris* pv. campestris B100	Wild type, Sm^r^	Hötte et al., 1990; Vorhölter et al., 2008 [5, 22]
Xcc H21012 (*wxcB*)	*wxcB*::Tn*5*-B20, Km^r^ [23], Sm^r^	Hötte et al., 1990; Vorhölter et al., 2001 [14, 22]
Xcc H28110 (*wxcK*)	*wxcK*::Tn*5*-B20, Km^r^ [23], Sm^r^	Hötte et al., 1990; Vorhölter et al., 2001 [14, 22]
Xcc H20110 (*wxcN*)	*wxcN*::Tn*5*-B20, Km^r^ [23], Sm^r^	Hötte et al., 1990; Vorhölter et al., 2001 [14, 22]

$$ RGR=\frac{\mathrm{In}(OD2)-\mathrm{In}(OD1)}{t2-t1} $$

### Glucose determination

Glucose was determined with the SuperGl ambulance device from Dr. Müller Gerätebau (Freital, Germany) and measurements were carried out in accordance to their description. 20 μl of 1/5 v/v sample in water was taken and mixed with 1000 μl of a haemolysis system solution (Hitado, Möhnesee, Germany). Samples were measured in triplicates for each time point.

### Nitrate determination

For nitrate determination 1/5 v/v sample in water was used and treated according to the instructions of the Merck Spectroquant nitrate kit (Merck Chemicals GmbH, Darmstadt, Germany). Photometric measurements were carried out at 340 nm.

### Xanthan and biomass determination

Xanthan and biomass determination was adapted from Palaniraj et al., 2011 [38]. Briefly, 5 ml of culture was harvested after 96 h of cultivation and diluted with water 1/8 v/v. This was centrifuged for 1 h at 12.500 rpm. The xanthan containing supernatant was separated from the pellet. The pellet was dried at 60 °C for biomass determination. Isopropanol was added to the supernatant 3/1 v/v and followed by 30 min chilling on ice and centrifugation for 1:30 h at 9.000 rpm. The resulting supernatant was discarded and the xanthan pellet was dried at 60 °C for 48 h.

### LPS isolation

LPS isolation was performed with cultures grown in TY media and harvested in the stationary phase. The LPS was isolated using the hot phenol / water method, described by Westphal and Jann [28].

### Methanolysis and peracetylation of LPS

100 μg of LPS were methanolyzed in 0.5 M HCl / MeOH for 45 min at 85 °C. After washing with methanol and drying, samples were peracetylated using pyridine:acetic anhydride 2/1 v/v for 30 min at 85 °C. Samples were washed with chloroform and then solved in chloroform for GC measurements.

### Hydrolysis, reduction and peracetylation of LPS

500 μg of LPS were hydrolyzed in 0.1 M HCl for 48 h at 100 °C. Then 15 μg of xylose was added as an internal standard and samples were dried with nitrogen. This was followed by three times washing with 10 % ether / hexane and then drying to remove fatty acids. Then the samples were reduced. They were dissolved in 0.5 ml water and the pH was adjusted to 8. Then three times 100 μl of a 10 mg*ml^−1^ NaBH_4_ solution were added. Samples were incubated overnight at room temperature in the dark. Afterwards some drops of 2 M HCl were added. Samples were dried and washed three times with 5 % acetic acid / MeOH, then samples were washed three times with methanol alone and dried. Peracetylation occurred with pydidine/acetic anhydride 1/1 v/v for 10 min at 85 °C. Samples were washed with chloroform and then dissolved in chloroform for GC measurements.

In order to determine the right amount of rhamnose inside the sample with the help of an internal standard, a response factor had to be determined. 20 μg of rhamnose and xylose were mixed and treated like the other samples. Through the peak areas a response factor could be calculated and for rhamnose this factor was 1.17.

### Gas Chromatography (GC) (Mass Spectrometry)

GC was started with an initial temperature of 150 °C for 3 min, then increased with a rate of 3 °C per minute until 250 °C and from there with 25 °C per minute until 320 °C for 10 min. Following column was used: Restek Rxi 5Sil MS column in 30 m. OA constituent rhamnose, as well as core moieties were detected via reference substances and FucNAc was detected with the help of the Mass Spectra analyzed using the Xcalibur software from Bruker (Bremen, Germany).

### Sample preparation for MALDI-MS analysis

For the MALDI-MS experiments 1,5-diamminonapthalene (DAN) was used as matrix. DAN solution (7 g/l) was prepared in acetonitrile. The LPS samples have been mixed with acetonitrile, 5 mg/ml. 1 μL of analyte and 1 μl of matrix were mixed, 1 μl of the analyte-matrix solution was spotted directly on the target plate (MTP Anchor Chip 384, Bruker, Bremen, Germany) and analyzed by MALDI-TOF-MS.

### MALDI measurements

Mass spectrometric analyses were performed in the linear negative mode accelerating potential on a time-of-flight mass spectrometer (Bruker Ultraflextreme III TOF-TOF; Bruker Daltonik, Bremen, Germany. Ion source 1 voltage -20.0 kV, ion source 2 voltage -18.8 kV, reflector 1 voltage 0 kV, reflector 2 voltage 0 kV and lens voltage -6 kV) which was equipped with a Smartbeam laser (Nd:YAG 355 nm) capable of operating at a repetition rate of 1000 Hz with optimized delayed extraction time, laser beam size was set to large and the number of shots was 500. Laser energy was optimized for signal-to-noise in each preparation. For every experiment, 20–50 spectra were accumulated and summarized.

## Additional files

Additional file 1: GC chromatograms of a rhamnose:xylose 1:1 (20 μg) standard, Xcc B100 wild type and OA mutant strains. Due to the standard peaks a response factor for rhamnose towards xylose could be calculated. B100 Wild type data were compared to the *wxcB* mutant H21012, *wxcK* mutant H28110 and *wxcN* mutant H20110 towards their rhamnose amounts, after hydrolysis and reduction of 500 μg LPS. Standard chromatogram is depicted as zoom to optimize the view. (PDF 1357 kb) Additional file 2: GC chromatogram replicates of Xcc B100 wild type and OA mutant strains. B100 Wild type data were compared to *wxcK* mutant H28110 and *wxcN* mutant H20110 towards their rhamnose amounts, after hydrolysis and reduction of 500 μg LPS. (PDF 1354 kb) Additional file 3: Cultivation replicates of the *Xanthomonas campestris* pv. campestris B100 wild type (A, B) the three mutants Xcc H21012 (*wxcB*) (C,D), Xcc H28110 (*wxcK*) (E,F) and Xcc H20110 (*wxcN*) (G,H). All strains were grown in XMD minimal media with 0.6 g/l KNO_3_ as nitrogen source and supplemented with 30 g/l glucose as carbon source, at 30 °C and 180 rpm. The cultivation occurred simultaneously under the same conditions for 96 h. Displayed are the culture titer (OD), glucose and nitrogen consumption over time. (PDF 1393 kb) Additional file 4: Determination of the growth rate of *Xanthomonas campestris* pv. campestris B100 wild type and mutants H21012 (*wxcB*), H28110 (*wxcK*) and H20110 (*wxcN*) (XLSX 9 kb) Additional file 5: Xanthan production and *t*-test of the *Xanthomonas campestris* pv. campestris B100 wild type and mutants H21012 (*wxcB*), H28110 (*wxcK*) and H20110 (*wxcN*). (XLSX 9 kb)

## Abbreviations

DAN: 1,5-diamminonapthalene
FucNAc: N-acetylfucosamine
GC: gas chromatography
GDP: guanosine di-phosphtate
Kdo: 3-deoxy-D-*manno*-octulonsonic acid
Km: kanamycin
LPS: lipopolysaccharide
MALDI: matrix-assisted laser desorption ionization
MeOH: methanol
MS: mass spectrometry
OA: o-antigen
OD: optical density
PAGE: polyacrylamid gel electrophoresis
SDS: sodium dodecyl sulfate
Sm: streptomycin
dTDP: thymidine di-phosphate
TOF: time of flight
UDP: uracil di-phosphate
Xcc: *Xanthomonas campestris* pathovar campestris
