# Supplementary figures and images for: The influence of a modified lipopolysaccharide O-antigen on the biosynthesis of xanthan in Xanthomonas campestris pv. campestris B100
Source: BMC Microbiol. 2016 May 23;16:93. doi: 10.1186/s12866-016-0710-y (PMC4878081; doi:10.1186/s12866-016-0710-y)

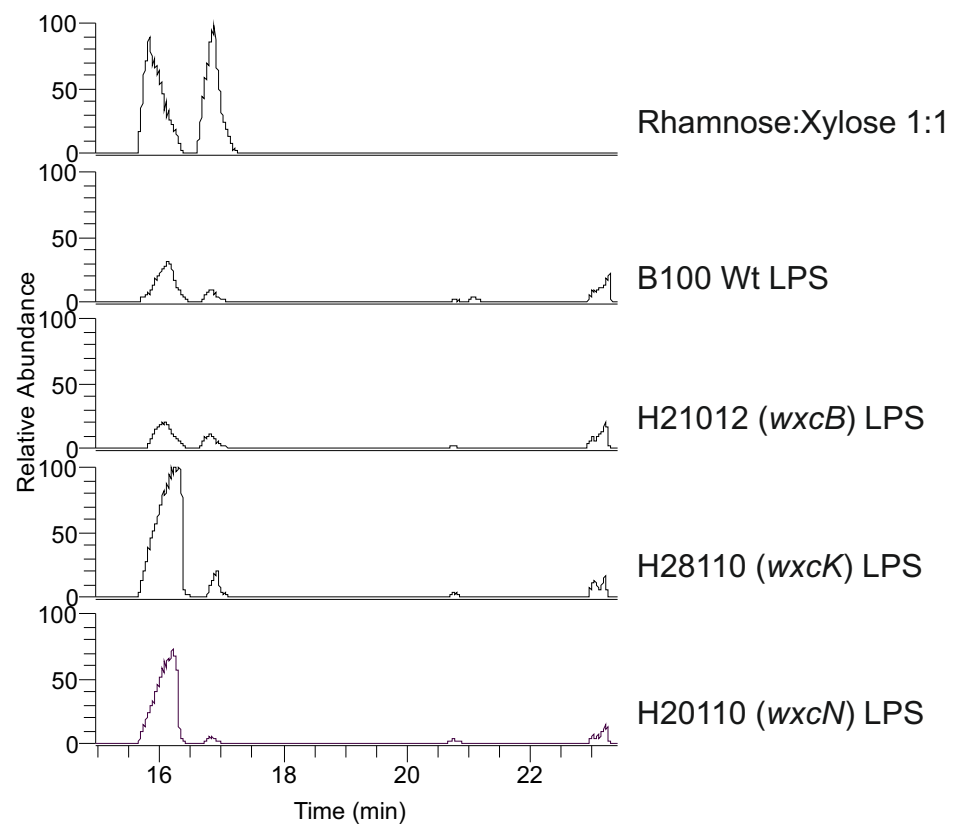

Supplement: Additional file 1: — GC chromatograms of a rhamnose:xylose 1:1 (20 μg) standard, Xcc B100 wild type and OA mutant strains. Due to the standard peaks a response factor for rhamnose towards xylose could be calculated. B100 Wild type data were compared to the wxcB mutant H21012, wxcK mutant H28110 and wxcN mutant H20110 towards their rhamnose amounts, after hydrolysis and reduction of 500 μg LPS. Standard chromatogram is depicted as zoom to optimize the view. (PDF 1357 kb) [file 12866_2016_710_MOESM1_ESM.pdf]

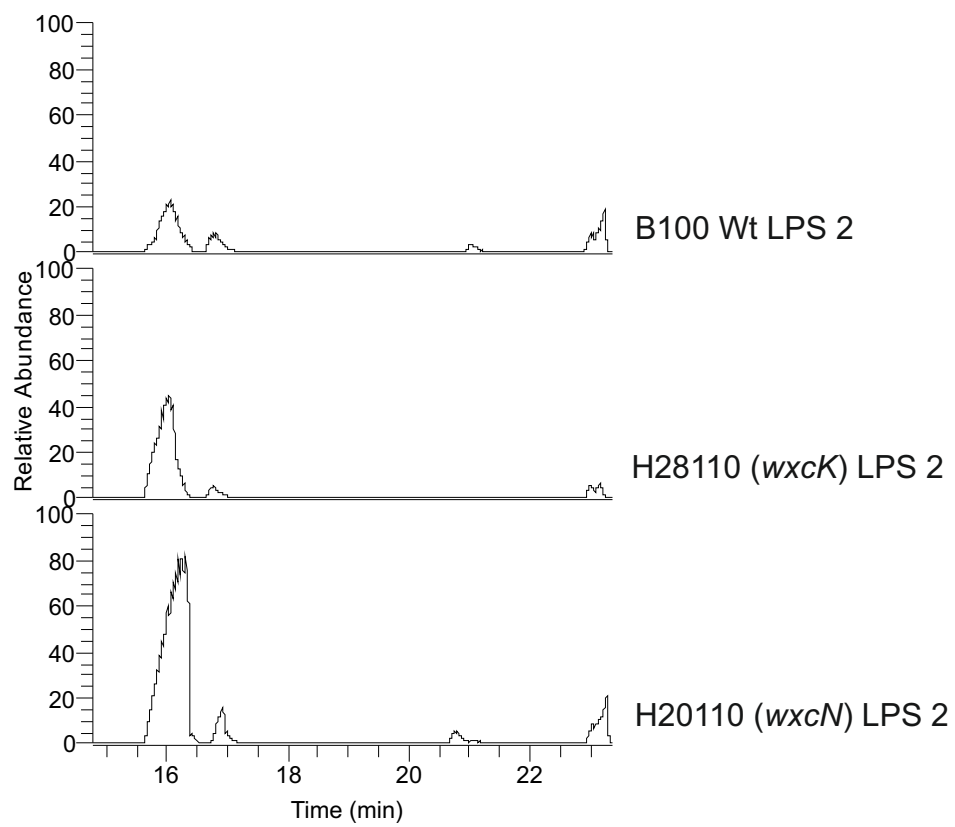

Supplement: Additional file 2: — GC chromatogram replicates of Xcc B100 wild type and OA mutant strains. B100 Wild type data were compared to wxcK mutant H28110 and wxcN mutant H20110 towards their rhamnose amounts, after hydrolysis and reduction of 500 μg LPS. (PDF 1354 kb) [file 12866_2016_710_MOESM2_ESM.pdf]

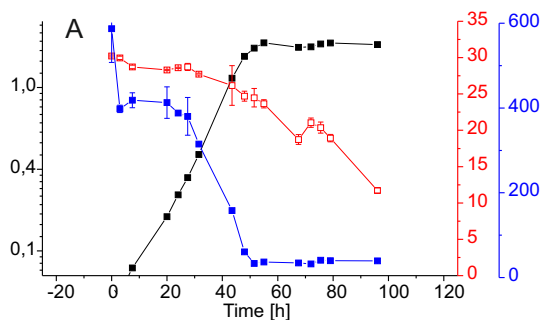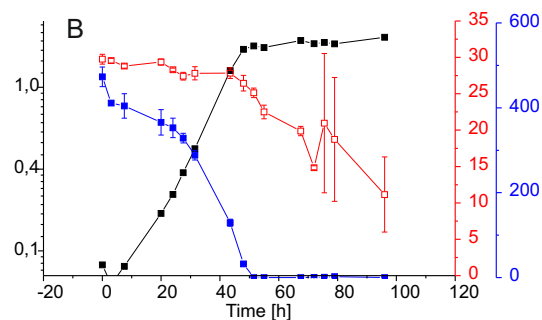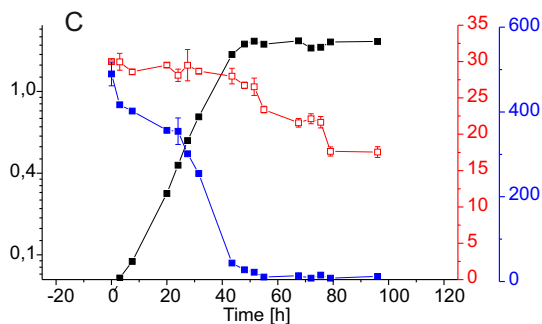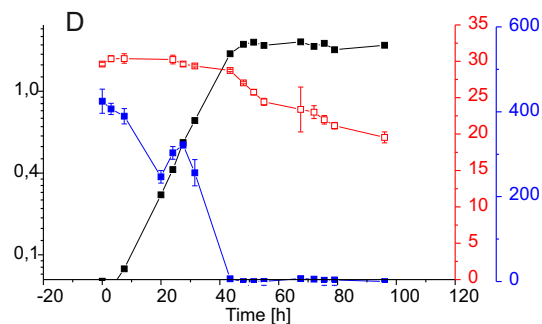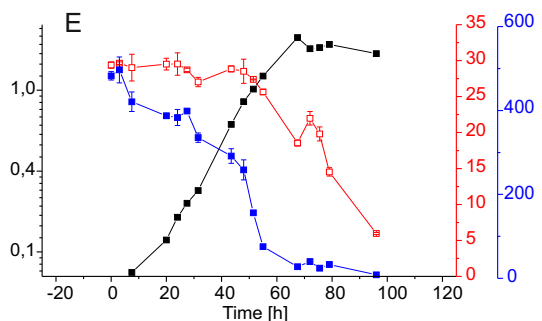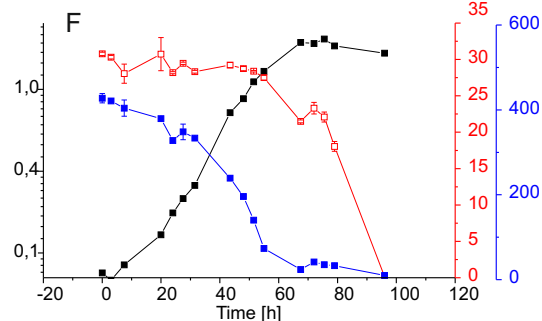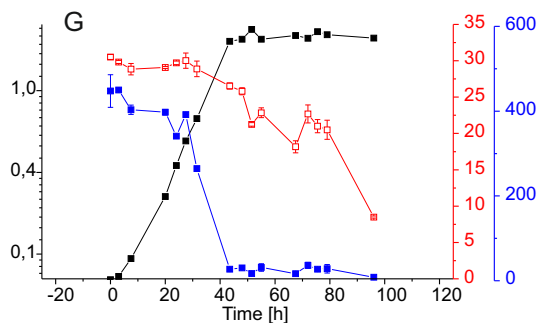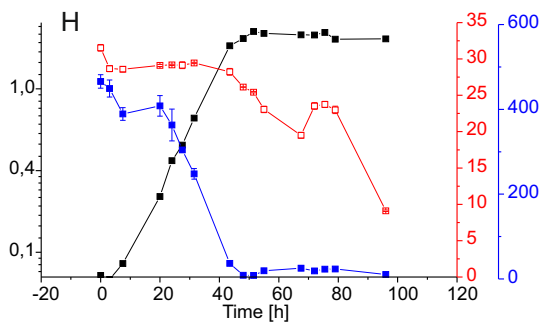

A: Wild type-2

B: Wild type-3

C: H21012 (*wxcB*)-2

D: H21012 (*wxcB*)-3

E: H28110 (*wxcK*)-2

F: H28110 (*wxcK*)-3

G: H20110 (*wxcN*)-2

H: H20110 (*wxcN*)-3

—■— OD [600]

—□— Glucose [g/l]

—■— Nitrate [mg/l]

Supplement: Additional file 3: — Cultivation replicates of the Xanthomonas campestris pv. campestris B100 wild type (A, B) the three mutants Xcc H21012 (wxcB) (C,D), Xcc H28110 (wxcK) (E,F) and Xcc H20110 (wxcN) (G,H). All strains were grown in XMD minimal media with 0.6 g/l KNO3 as nitrogen source and supplemented with 30 g/l glucose as carbon source, at 30 °C and 180 rpm. The cultivation occurred simultaneously under the same conditions for 96 h. Displayed are the culture titer (OD), glucose and nitrogen consumption over time. (PDF 1393 kb) [file 12866_2016_710_MOESM3_ESM.pdf]
